# Evaluation of sulbactam/durlobactam activity and synergy against highly drug-resistant *Acinetobacter baumannii* strains

**DOI:** 10.1093/jacamr/dlaf220

**Published:** 2025-11-19

**Authors:** Justin Halim, Jeannete Bouzo, Valerie J Carabetta

**Affiliations:** Department of Biomedical Sciences, Cooper Medical School of Rowan University, Camden, NJ 08103, USA; Rowan-Virtua School of Osteopathic Medicine, Stratford, NJ 08084, USA; Department of Biomedical Sciences, Cooper Medical School of Rowan University, Camden, NJ 08103, USA

## Abstract

**Background:**

*Acinetobacter baumannii* is a bacterial pathogen frequently implicated in healthcare-associated infections, with limited effective treatment options due to widespread antibiotic resistance. Sulbactam/durlobactam is a novel β-lactam/β-lactamase inhibitor (βL/βLI) combination recently approved for the treatment of hospital-associated bacterial pneumonia and ventilator-associated bacterial pneumonia due to *A. baumannii*.

**Methods:**

We evaluated the *in vitro* activity of sulbactam/durlobactam alone and in combination with 15 clinically relevant antibiotics against 22 *A. baumannii* clinical isolates, including 21 extensively or pandrug-resistant strains (M1–M22) and one metallo-β-lactamase (MBL)-producing strain (BAA-3302). Susceptibility testing, chequerboard synergy assays, and static time-kill assays were performed to assess antimicrobial interactions.

**Results:**

All 21 XDR/PDR isolates were susceptible to sulbactam/durlobactam (MIC < 4/4 mg/L), while the MBL-harbouring strain BAA-3302 showed intermediate susceptibility (MIC = 8/4 mg/L). Chequerboard assays revealed consistent synergy between sulbactam/durlobactam and multiple β-lactams and βL/βLI agents, including cefepime, meropenem, cefiderocol, ceftazidime–avibactam, and piperacillin–tazobactam, with ≥95% of strains showing synergistic effects. Selected combinations demonstrated rapid and sustained bactericidal activity against select strains in time-kill assays. Combinations with tetracyclines also showed synergy in select strains, particularly against the MBL-carrying isolate.

**Conclusions:**

Sulbactam/durlobactam demonstrates strong activity against highly drug-resistant *A. baumannii* isolates and shows enhanced potency when combined with specific β-lactam and βL/βLI agents. Further investigation of sulbactam/durlobactam-based combination therapy is warranted as a therapeutic strategy for the treatment of carbapenem-resistant *A. baumannii* infections.

## Introduction

*Acinetobacter baumannii* is a Gram-negative opportunistic pathogen frequently implicated in healthcare-associated infections, especially in critically ill or immunocompromised patients.^1,2^ Despite its relatively low intrinsic virulence, infections can result in high mortality, particularly when caused by drug-resistant strains.^3,4^ The rapid emergence of multidrug-resistant (MDR), extensively drug-resistant (XDR), and pandrug-resistant (PDR) *A. baumannii* strains—including carbapenem-resistant *A. baumannii* (CRAB)—has severely limited treatment options and prompted its designation by the CDC as an urgent public health threat.^5–9^

Sulbactam–durlobactam is a recently FDA-approved β-lactam/β-lactamase inhibitor (βL/βLI) combination specifically labelled for the treatment of hospital-acquired bacterial pneumonia and ventilator-associated bacterial pneumonia (HABP/VABP) caused by susceptible isolates of *A. baumannii–calcoaceticus* complex.^10^ Sulbactam exerts intrinsic antibacterial activity against *A. baumannii* by targeting penicillin-binding proteins (PBPs), while durlobactam protects sulbactam from degradation through broad-spectrum inhibition of Ambler Class A, C, and D β-lactamases.^10,11^ Based on favourable clinical outcomes in the ATTACK trial, sulbactam/durlobactam is now recommended as first-line treatment for CRAB.^12,13^ However, resistance mechanisms against sulbactam/durlobactam, primarily via Class B metallo-β-lactamases (MBLs) and PBP mutations, have been identified and underscore the need to optimize and preserve sulbactam/durlobactam efficacy.^14,15^ Combination antibiotic therapy represents a promising strategy to enhance antimicrobial activity and reduce resistance selection.^16^ A prior study reported *in vitro* synergy between sulbactam/durlobactam and cefepime,^17^ and we previously reported *in vitro* synergy between sulbactam/durlobactam and cefiderocol.^18^ However, comprehensive evaluations across diverse drug-resistant isolates remain limited. In this study, we assessed the *in vitro* activity of sulbactam/durlobactam as monotherapy and in combination with 15 antibiotics against 21 XDR/PDR clinical isolates and one NDM-producing strain of *A. baumannii*, with the aim of identifying novel synergistic or additive combinations.

## Methods

### Bacterial strains, media, and growth conditions

The 21 *A. baumannii* isolates (M1–M22) were collected from Cooper University Hospital (Camden, NJ, USA). All isolates were de-identified to protect patient privacy; therefore, detailed clinical source data (e.g. infection site, patient demographics) are not available. Prior genomic analyses demonstrated that the isolates are not clonally related and harbour distinct resistance determinants, including class C and D β-lactamases.^19^ Of the original 22 isolates collected, one strain (M15) was excluded from further study due to contamination during initial characterization, as previously reported.^19^ Therefore, isolates are numbered M1–M22, excluding M15. An additional strain BAA-3302, a metallo-β-lactamase (MBL)-carrying strain, was obtained from the American Type Culture Collection (ATCC). Mueller Hinton broth (MHB) and iron-depleted, cation-adjusted Mueller Hinton broth (ID-CAMHB) were prepared as described previously.^20^ Bacterial strains were inoculated into MHB and grown overnight in a 37°C incubator with shaking. Bacterial growth was then assessed by measuring the optical density at 600 nm (OD_600_). Durlobactam was provided by Innoviva Specialty Therapeutics, Inc. (Waltham, MA, USA). Cefiderocol was provided by Shionogi (Florham Park, NJ, USA). Omadacycline was provided by Paratek Pharmaceuticals (Boston, MA, USA). All other antibiotics were purchased as previously described.^21^

### Determination of the MIC

MIC values for sulbactam/durlobactam were determined by broth microdilution using standard protocols from the American Society for Microbiology and the Clinical and Laboratory Standards Institute (CLSI).^22^ Overnight cultures were diluted in MHB to an OD_600_ value of 0.05. Sulbactam was added to the first well of a 96-well plate at 2× the starting concentration (32 mg/L), followed by 2-fold serial dilutions. Equal volumes of diluted bacterial cells were added to each well. Durlobactam was added at a fixed concentration of 4 mg/L per CLSI recommendations. Plates were incubated overnight at 37°C without shaking, and OD_600_ was measured the next day using a Synergy H1 Microplate reader (Biotek). The MIC was determined as the lowest concentration of drug that inhibited bacterial growth, defined by an OD_600_ < 0.1, which was our cut-off for growth inhibition. MICs were determined from at least two independent experiments. CLSI breakpoints were used to interpret susceptibility to sulbactam/durlobactam (≤4/4 susceptible; 8/4 intermediate; ≥16/4 resistant).^14,21^ Previously, we found that MICs were 2- to 16-fold lower in ID-CAMHB.^18^ These MIC values were used to guide appropriate antibiotic starting concentrations for dilution to perform chequerboard assays, including cefiderocol.

### Chequerboard assays

Chequerboard assays were performed as described previously.^22^ Two-fold serial dilutions of sulbactam and a second antibiotic were prepared in perpendicular directions in 96-well plates. Antibiotics were selected based on prior studies^18^ and added at 4× starting concentrations, consistent with earlier protocols.^22^ Chequerboard assays were initially performed using a starting sulbactam concentration of 32 mg/L, but given the consistently low MIC values observed, the starting concentration was reduced to 4 mg/L in subsequent assays to better resolve synergy effects. Antibiotics were selected based on prior reports of potential synergy with β-lactam/β-lactamase inhibitor combinations, including our previous work,^18,19^ as well as clinical relevance for the treatment of multidrug-resistant *A. baumannii*.^17^ This panel encompassed β-lactams and βL/βLI agents cefepime, ceftriaxone, ceftazidime, meropenem, cefiderocol, ceftazidime/avibactam, and piperacillin/tazobactam; tetracyclines eravacycline, omadacycline, minocycline, doxycycline; aminoglycosides amikacin, tobramycin, ciprofloxacin, and rifampin. For ceftazidime/avibactam and piperacillin/tazobactam, ceftazidime and piperacillin were serially diluted, while avibactam and tazobactam were added at a constant 4 mg/L. All chequerboard assays evaluating cefiderocol were conducted in ID-CAMHB. Diluted bacterial cultures (OD_600_ = 0.05) were added, and plates were incubated overnight. OD_600_ readings were taken the next morning. Synergy was assessed using the fractional inhibitory concentration index (FICI), calculated as the sum of each drug’s concentration in combination divided by its MIC alone. The FICI was then calculated using the following formula: FICI = (MIC A_A+B_/MIC A) + (MIC B_A+B_/MIC B), in which MIC A and MIC B denote the MIC value of each antibiotic alone and MIC A_A+B_ and MIC B_A+B_ denote the MIC values of the drugs in combination. For a FICI of ≤0.5, the combination is synergistic; for a FICI >0.5 and ≤1.0, the combination is additive; for a FICI >1 and <4, there is no effect; for a FICI ≥4, the interaction is antagonistic. All determinations were made at least two independent times.

### Static time-kill assays

Select antibiotic combinations showing high synergy with sulbactam/durlobactam in chequerboard assays were further evaluated using static time-kill assays (TKAs) to confirm bactericidal activity. Strains M1, M20, and BAA-3302 were tested in MHB (or ID-CAMHB for cefiderocol-containing assays) at an initial inoculum of ∼10^6^ CFU/mL. Paired antibiotics included cefepime, meropenem, cefiderocol, ceftazidime/avibactam, piperacillin/tazobactam, and eravacycline. TKAs were performed under four conditions: (i) untreated control, (ii) sulbactam/durlobactam alone, (iii) comparator antibiotic alone, and (iv) sulbactam/durlobactam plus comparator antibiotic. All agents were added at 0.5× MIC. Assays were carried out in 5-mL volumes at 37°C with shaking. Samples were taken at 0, 2, 4, 8, and 24 h, serially diluted, and drop-plated (10 μL, in triplicate) on Luria–Bertani (LB) agar. Plates were incubated at 37°C for 12–16 h. Experiments were performed in biological duplicate, and mean CFU/mL values were calculated with error bars indicating standard deviation. Synergy was defined as a ≥ 2 log_10_ CFU/mL reduction at 24 h versus the most active single agent; additivity as a 1 to <2 log_10_ reduction; and indifference as <1 log_10_ reduction. TKA curves were generated using GraphPad Prism version 10.1.5 (GraphPad Software, San Diego, CA, USA).

### Whole-genome sequencing and bioinformatics

Genomic DNA was isolated as previously described.^19^ Genomic DNA samples were sent to Genewiz (South Plainfield, NJ) for whole-genome sequencing. Paired-end sequencing reads (150 bp) were adapter-trimmed and quality-filtered with the fastp toolkit. Human background was removed using Snap-aligner by mapping the reads to a set of human and chimpanzee reference genomes. Microbial reads were assembled with MEGAHIT, and contigs were analysed with AMRFinderPlus (ver. 3.11) for the presence of antimicrobial resistance genes.

## Results

### Determination of susceptibilities of *A. baumannii* clinical isolates to sulbactam/durlobactam

We previously tested our collection of clinical isolates (M1–M22) against standard-of-care antibiotics and found that all were XDR or PDR, with strains M5, M9, M17, M19, M20, and M21 classified as PDR, based on traditional definitions.^19^ To characterize their susceptibilities to sulbactam/durlobactam, standard broth microdilution assays were conducted in MHB to determine MIC values (Table 1). All 21 original strains were susceptible to sulbactam/durlobactam, as defined by an MIC value ≤4/4 mg/L. Strain BAA-3302 had intermediate susceptibility (MIC = 8/4 mg/L) and was the only non-susceptible strain. Whole-genome sequencing of 15 representative isolates, including 7 we previously reported,^18,19^ identified a diverse repertoire of β-lactam resistance mechanisms encompassing class C and D β-lactamases, PBP3 mutations, and efflux pumps (Table S1, available as Supplementary data at *JAC-AMR* Online). Notably, all 21 original isolates (M1-M22) were susceptible to sulbactam/durlobactam, underscoring its broad potency despite diverse resistance mechanisms.

**Table 1.** Antibiotic susceptibilities of each *A. baumannii* isolate to sulbactam/durlobactam (SUL/DUR)

Strain	SUL/DUR MIC (mg/L) Replicate 1	SUL/DUR MIC (mg/L) Replicate 2	S/I/R	Strain	SUL/DUR MIC (mg/L) Replicate 1	SUL/DUR MIC (mg/L) Replicate 2	S/I/R
**M1**	2/4	4/4	S	**M12**	2/4	2/4	S
**M2**	1/4	1/4	S	**M13**	2/4	2/4	S
**M3**	1/4	1/4	S	**M14**	1/4	2/4	S
**M4**	1/4	1/4	S	**M16**	0.5/4	1/4	S
**M5**	1/4	1/4	S	**M17**	1/4	2/4	S
**M6**	1/4	1/4	S	**M18**	2/4	2/4	S
**M7**	0.5/4	0.5/4	S	**M19**	2/4	2/4	S
**M8**	0.5/4	0.5/4	S	**M20**	2/4	2/4	S
**M9**	1/4	2/4	S	**M21**	2/4	2/4	S
**M10**	2/4	2/4	S	**M22**	2/4	2/4	S
**M11**	0.25/4	0.5/4	S	**BAA-3302**	8/4	8/4	I

Strains from our collection are labelled M1–M22. Note that strain M15 was excluded from the study due to contamination. MIC values were determined at least two independent times, with both values presented. Strains that are susceptible are denoted as S; strains with intermediate susceptibility are denoted as I; strains that are resistant are denoted as R, according to CLSI standards.

### Determination of combinatorial effects between sulbactam/durlobactam and various antibiotics

**Figure 1.**
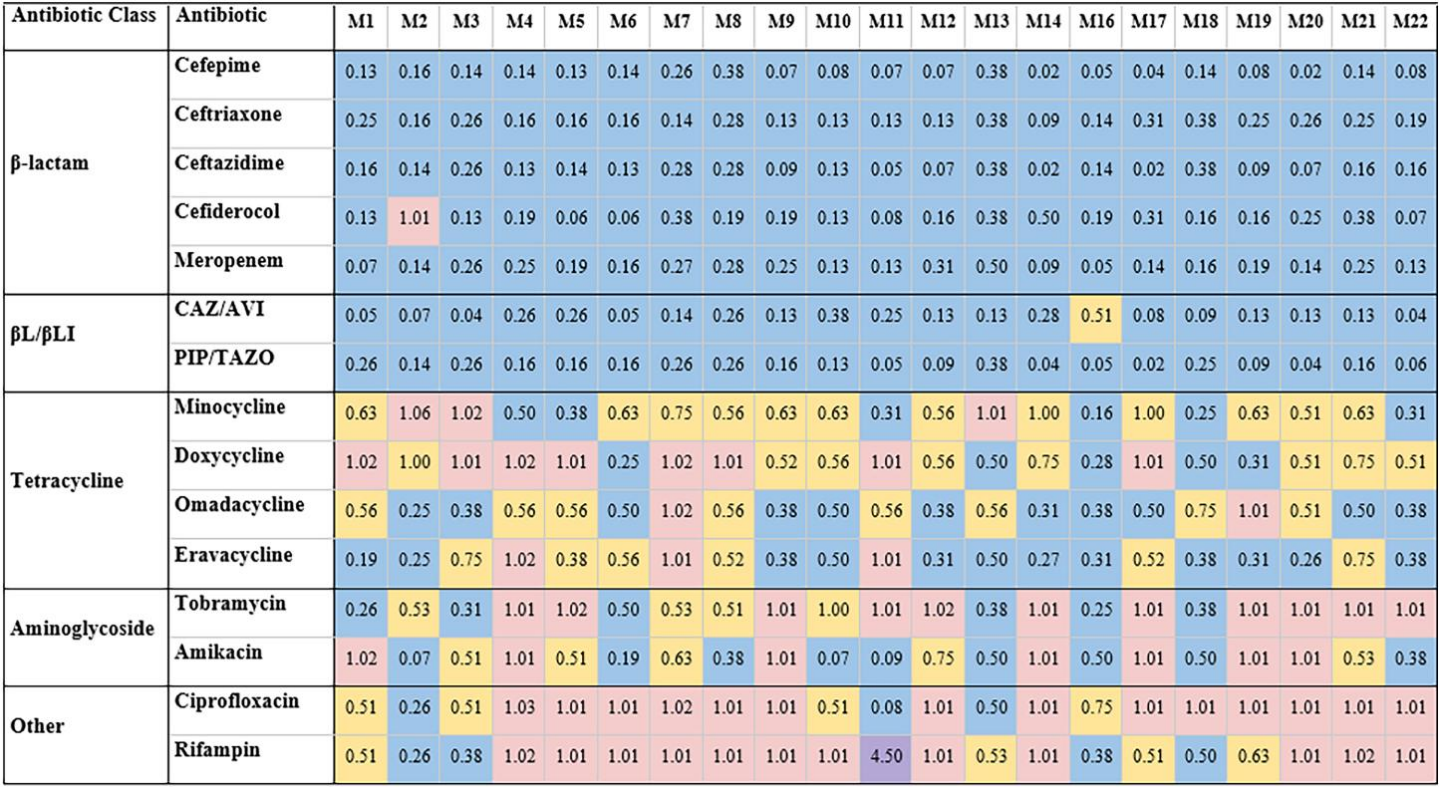
Fractional inhibitory concentration index (FICI) values obtained from sulbactam/durlobactam in combination with various antibiotics against *A. baumannii* strains M1–M22. FICI values in the synergistic range (≤0.5) are reported in blue; FICI values in the additive range (0.5–1.0) are reported in yellow; FICI values indicating no interaction (1.0–4.0) are reported in pink; FICI values in the antagonistic range (>4.0) are reported in purple. PIP/TAZO, piperacillin/tazobactam; CAZ/AVI, ceftazidime/avibactam.

We next determined if any antibiotics combined with sulbactam/durlobactam are more potent in combination than was observed with each agent individually. Representative antibiotics from multiple drug classes were selected for a total of 15 combinations. We performed chequerboard assays for sulbactam/durlobactam in combination with each of these antibiotics against all strains to assess for combinatorial interactions (Figure 1). Utilization of clinically relevant concentrations at or above the MIC resulted in no growth of these XDR/PDR isolates when exposed to sulbactam/durlobactam alone. As a result of this potency, the synergistic potential of sulbactam/durlobactam was only recognized when utilizing a fraction of the MIC. The fractional inhibitory concentration (FIC) values for each pair were measured, and FICI values were then calculated to determine whether an interaction was synergistic, additive, indifferent, or antagonistic. FICI values calculated for each combination against each strain are shown in Figure 1. Rates of combinatorial interactions for each antibiotic combined with sulbactam/durlobactam are shown in Table 2.

**Table 2.** Rates of synergistic, additive, antagonistic, or lack of effects between antibiotics paired with sulbactam/durlobactam

Paired antibiotic	Synergistic	Additive	No interaction	Antagonistic
**Cefepime**	100.0%	0.0%	0.0%	0.0%
**Ceftriaxone**	100.0%	0.0%	0.0%	0.0%
**Ceftazidime**	100.0%	0.0%	0.0%	0.0%
**Cefiderocol**	95.2%	0.0%	4.8%	0.0%
**Meropenem**	100.0%	0.0%	0.0%	0.0%
**Ceftazidime/avibactam**	95.2%	4.8%	0.0%	0.0%
**Piperacillin/tazobactam**	100.0%	0.0%	0.0%	0.0%
**Minocycline**	28.6%	57.1%	14.3%	0.0%
**Doxycycline**	23.8%	38.1%	38.1%	0.0%
**Omadacycline**	52.4%	38.1%	9.5%	0.0%
**Eravacycline**	61.9%	23.8%	14.3%	0.0%
**Tobramycin**	28.6%	19.0%	52.4%	0.0%
**Amikacin**	42.9%	23.8%	33.3%	0.0%
**Ciprofloxacin**	14.3%	19.0%	66.7%	0.0%
**Rifampin**	19.0%	19.0%	57.2%	4.8%

**Figure 2.**
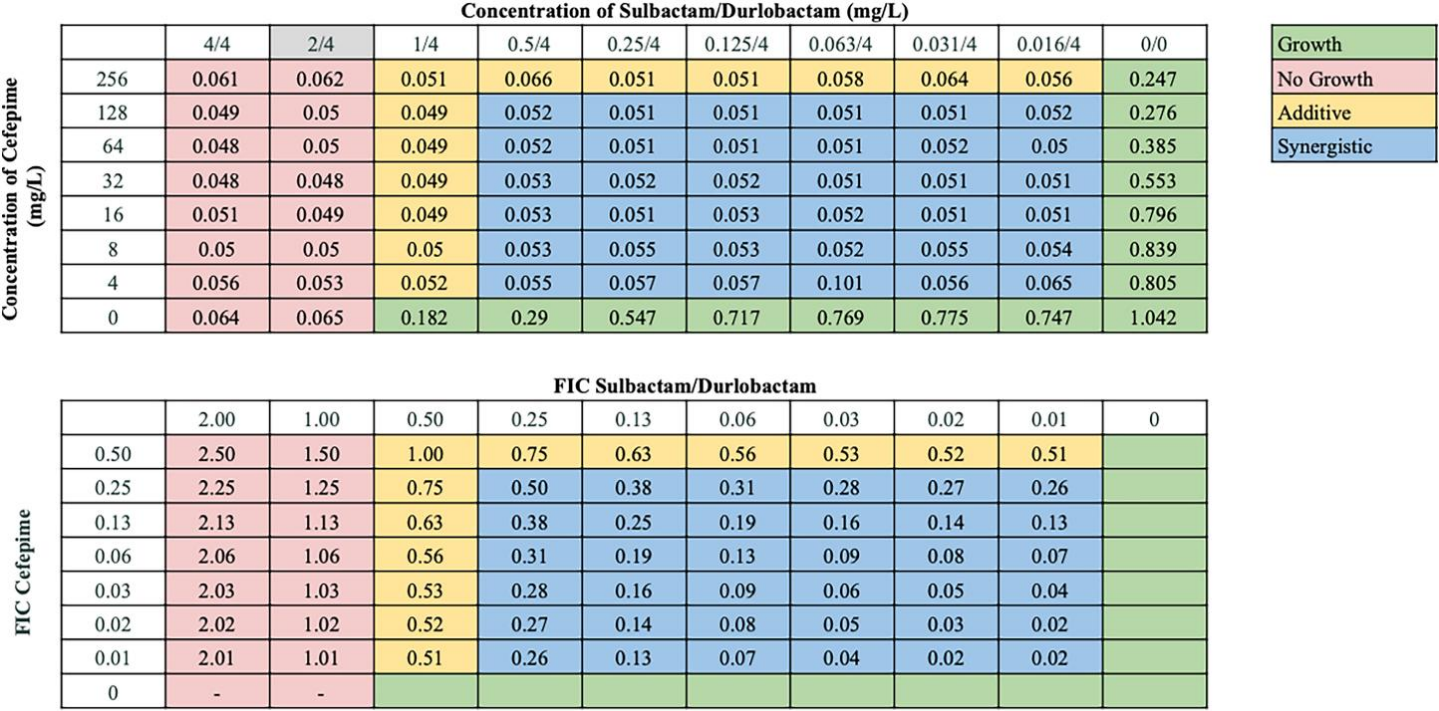
Representative chequerboard assay with sulbactam/durlobactam and cefepime against strain M20. Top: OD_600_ measurements following 16 h of static growth at 37°C. The MIC values for each drug alone are highlighted. If the MIC value exceeded the initial concentration, then no value is highlighted. No bacterial growth occurred in wells where OD_600_ < 0.1, and above this cutoff, bacterial growth did occur. Bottom: Fractional inhibitory concentration (FIC) values were calculated for each drug (concentration/MIC) and added together for all wells where no growth was observed. Additive interactions (FICI between 0.5–1.0), and synergistic interactions (FICI  ≤ 0.5) are indicated.

**Figure 3.**
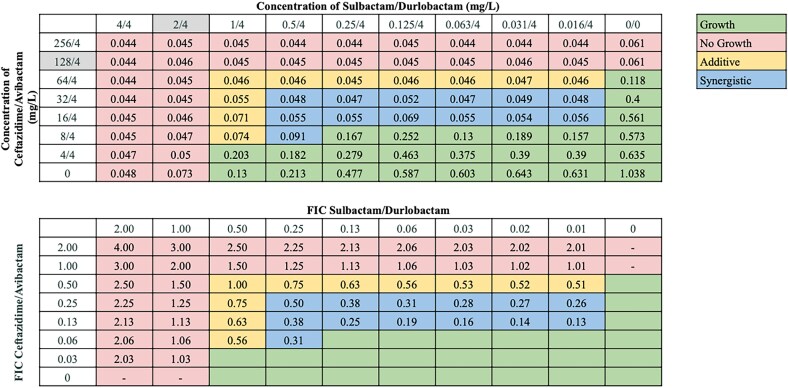
Representative chequerboard assay with sulbactam/durlobactam and ceftazidime/avibactam against strain M20. Format and interpretation as in Figure 2.

**Figure 4.**
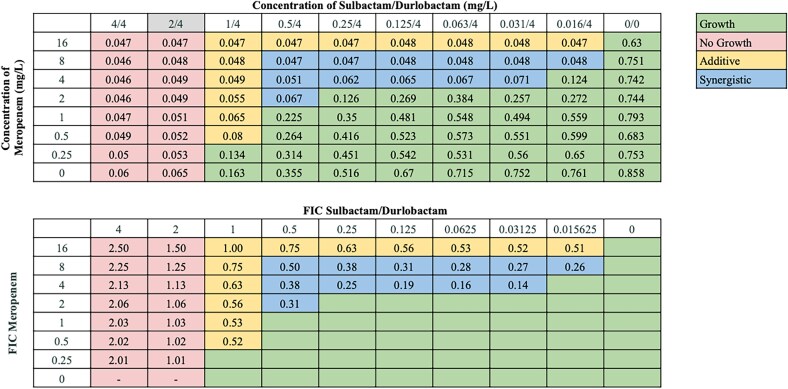
Representative chequerboard assay with sulbactam/durlobactam and meropenem against strain M20. Format and interpretation as in Figure 2.

Synergistic interactions between sulbactam/durlobactam and other antibiotics varied across strains, consistent with our previous observations.^18,19^ Sulbactam/durlobactam consistently demonstrated synergy with β-lactams and βL/βLIs, including cefepime, ceftriaxone, ceftazidime, meropenem, and piperacillin/tazobactam across all isolates, and with cefiderocol and ceftazidime/avibactam in almost all isolates. Despite high baseline resistance to most β-lactams in our isolate panel (excluding cefiderocol),^18,19^ their addition substantially reduced sulbactam/durlobactam MICs—up to 128-fold in some strains. Cefepime consistently produced the lowest FICI values across numerous strains (Figure 1), and representative chequerboard assays for key β-lactam combinations with sulbactam/durlobactam are shown in Figures 2–4 and Figures S1–S4.

**Figure 5.**
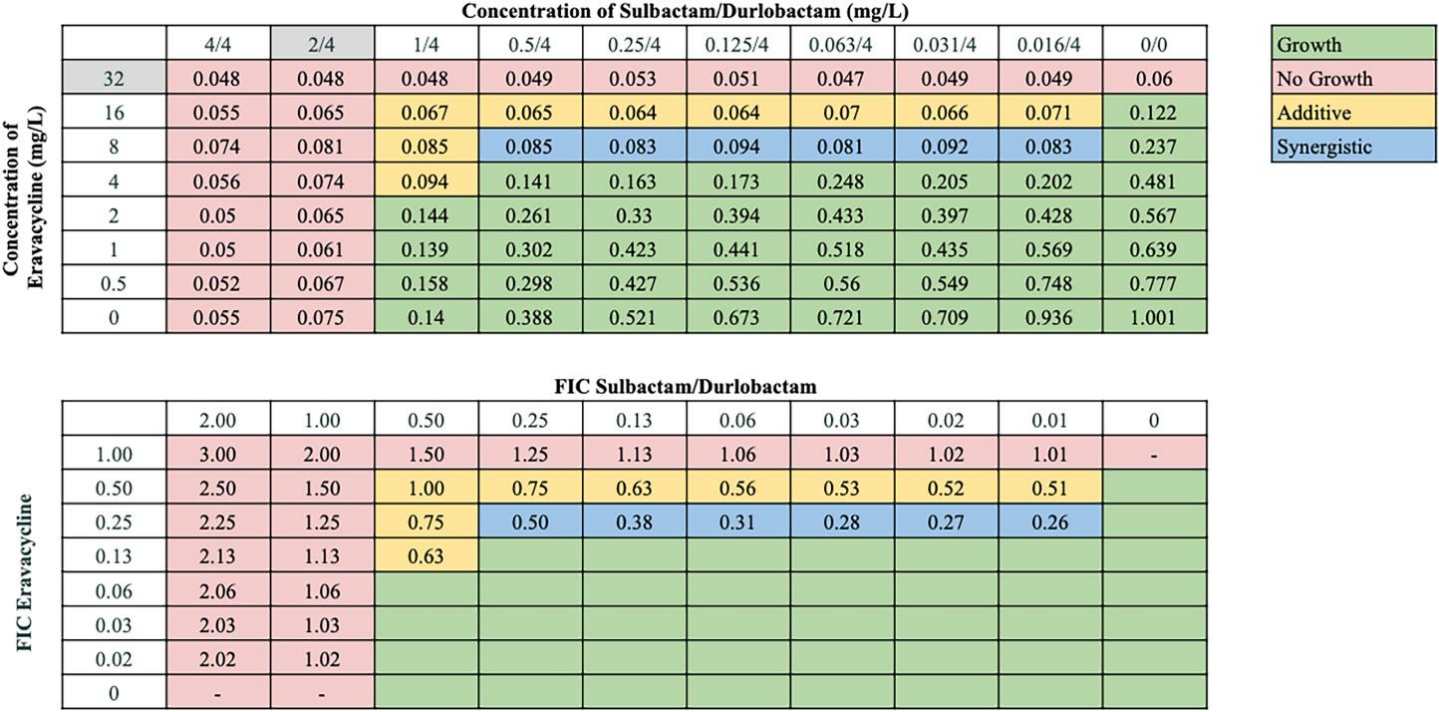
Representative chequerboard assay with sulbactam/durlobactam and eravacycline against strain M20. Format and interpretation as in Figure 2.

Among the tetracyclines, sulbactam/durlobactam demonstrated consistent synergy with eravacycline and omadacycline across most isolates (61.9% and 52.4%, respectively), while synergy with minocycline and doxycycline was less frequently demonstrated (Table 2, Figure 5, and Figure S5). Synergy was also common with the aminoglycosides amikacin and tobramycin (42.9% and 28.6%, respectively), though not universally observed. In contrast, synergy with ciprofloxacin and rifampin was infrequent (14.3% and 19.0%, respectively), with most isolates showing additive or indifferent effects. Rifampin exhibited antagonism with sulbactam/durlobactam against a single strain, as shown in the representative assay in Figure S6.

### Determination of combinatorial effects against an MBL-carrying strain

**Figure 6.**
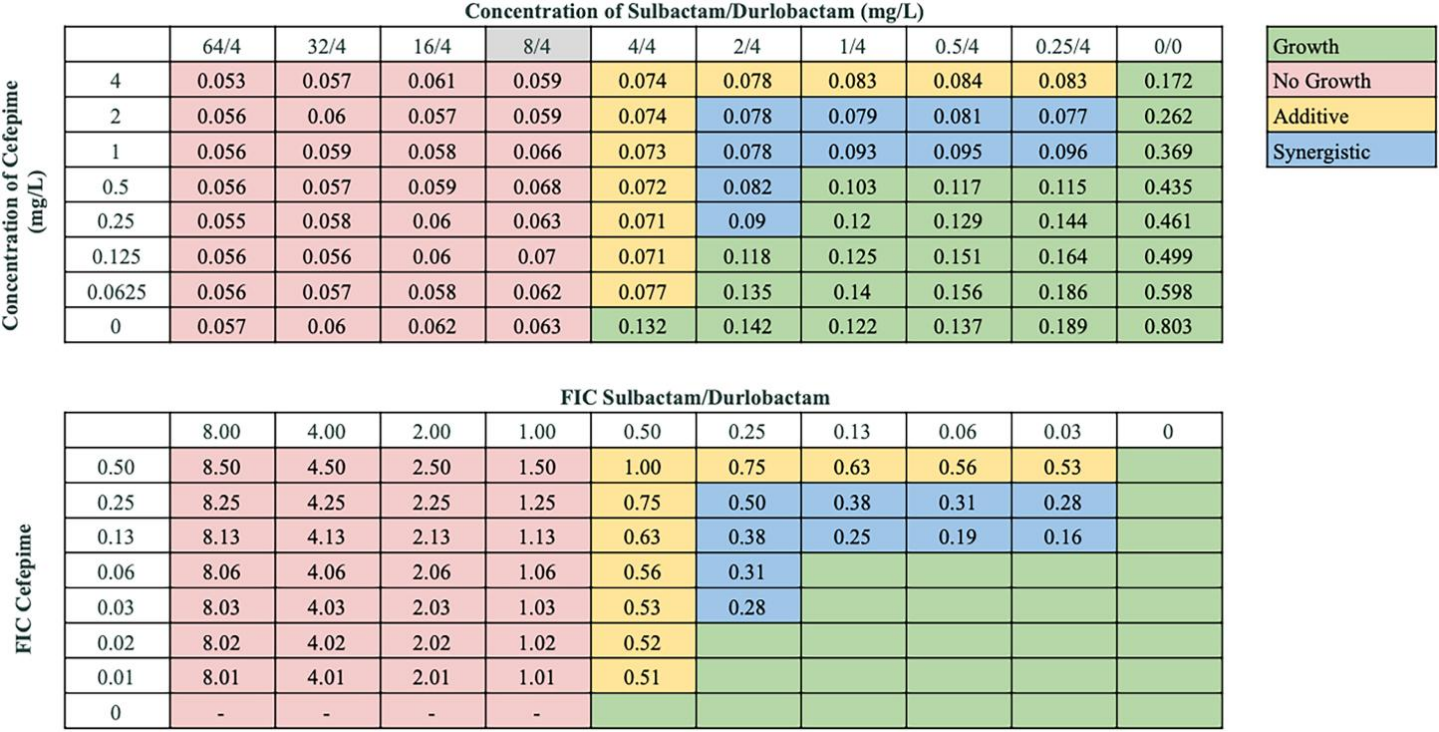
Representative chequerboard assay with sulbactam/durlobactam and cefepime against strain BAA-3302. Format and interpretation as in Figure 2.

**Figure 7.**
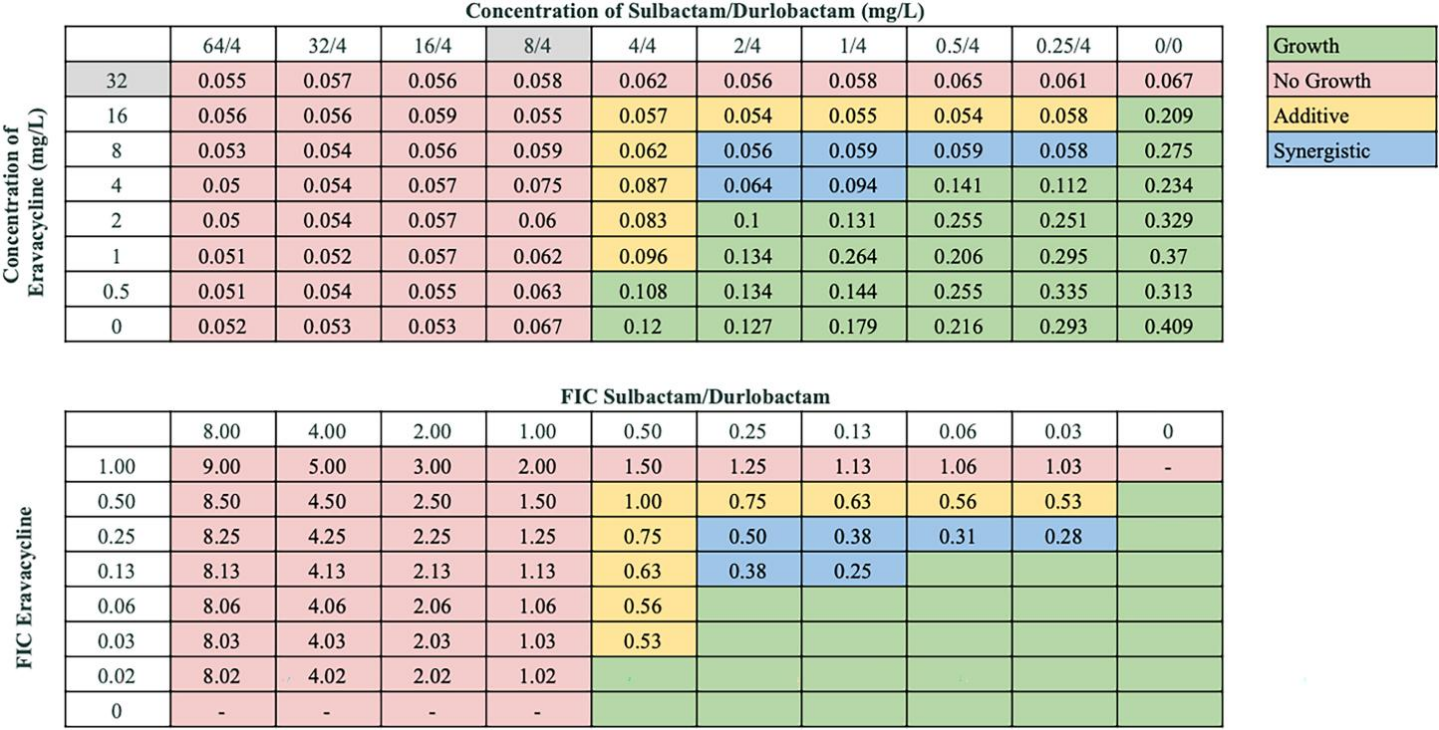
Representative chequerboard assay with sulbactam/durlobactam and eravacycline against strain BAA-3302. Format and interpretation as in Figure 2.

As none of our collection of 21 strains were known to possess MBLs, we tested these antibiotic combinations against strain BAA-3302, an MBL-carrying clinical isolate non-susceptible to sulbactam/durlobactam, with an MIC of 8/4 mg/L (Table 3). Overall, fewer β-lactam combinations retained synergy (cefepime, meropenem, piperacillin/tazobactam, cefiderocol), while others demonstrated only additive effects (ceftriaxone, ceftazidime, ceftazidime/avibactam). A representative chequerboard showing synergy between sulbactam/durlobactam and cefepime against BAA-3302 is shown in Figure 6. However, all tetracyclines (minocycline, doxycycline, omadacycline, and eravacycline) synergized with sulbactam/durlobactam against BAA-3302. A representative chequerboard showing synergy between sulbactam/durlobactam and eravacycline against BAA-3302 is shown in Figure 7. No interactions were observed with sulbactam/durlobactam combined with aminoglycosides tobramycin or amikacin. Additionally, ciprofloxacin synergized with sulbactam/durlobactam, while rifampin demonstrated additivity.

**Table 3.** Combinatorial effects of sulbactam/durlobactam -containing antibiotic combinations against strain BAA-3302, an MBL-harbouring strain non-susceptible to sulbactam/durlobactam (MIC = 8/4 mg/L)

Paired antibiotic	FICI Value	Interpretation
**Cefepime**	**0**.**16**	**Synergy**
Ceftriaxone	0.75	Additive
Ceftazidime	0.75	Additive
Ceftazidime/avibactam	0.56	Additive
**Cefiderocol**	0.19	**Synergy**
**Meropenem**	0.38	**Synergy**
**Piperacillin/tazobactam**	0.50	**Synergy**
**Minocycline**	0.19	**Synergy**
**Doxycycline**	0.16	**Synergy**
**Omadacycline**	0.16	**Synergy**
**Eravacycline**	0.25	**Synergy**
Tobramycin	1.01	No Interaction
Amikacin	1.01	No Interaction
**Ciprofloxacin**	0.38	**Synergy**
Rifampin	0.53	Additive

FICI values are reported in addition to interaction interpretation. Paired antibiotics in bold displayed synergistic interactions with sulbactam/durlobactam.

### Confirmation of bactericidal synergy against select strains with time-kill assays

We next confirmed bactericidal synergy among certain sulbactam/durlobactam-containing combinations through static TKAs against three representative isolates: M1 and M20, which are two PDR clinical isolates susceptible to sulbactam/durlobactam but with relatively higher MIC values (2–4 mg/L),^18,19^ and BAA-3302, an MBL-producing isolate with intermediate susceptibility to sulbactam/durlobactam (8/4 mg/L). These strains were selected to capture diverse and clinically relevant resistance phenotypes for further evaluation of bactericidal synergy. Among the combinations that demonstrated high synergy rates in chequerboard assays, we selected sulbactam/durlobactam in combination with cefepime, meropenem, cefiderocol, ceftazidime/avibactam, piperacillin/tazobactam, and eravacycline for TKA analysis. These agents represent diverse and clinically relevant antibiotic classes, extended-spectrum cephalosporins (cefepime and cefiderocol), carbapenems (meropenem), βL/βLI combinations (ceftazidime/avibactam and piperacillin/tazobactam), and tetracyclines (eravacycline). All agents were added at 0.5× MIC level to better evaluate synergy effects (Table S2).

**Figure 8.**
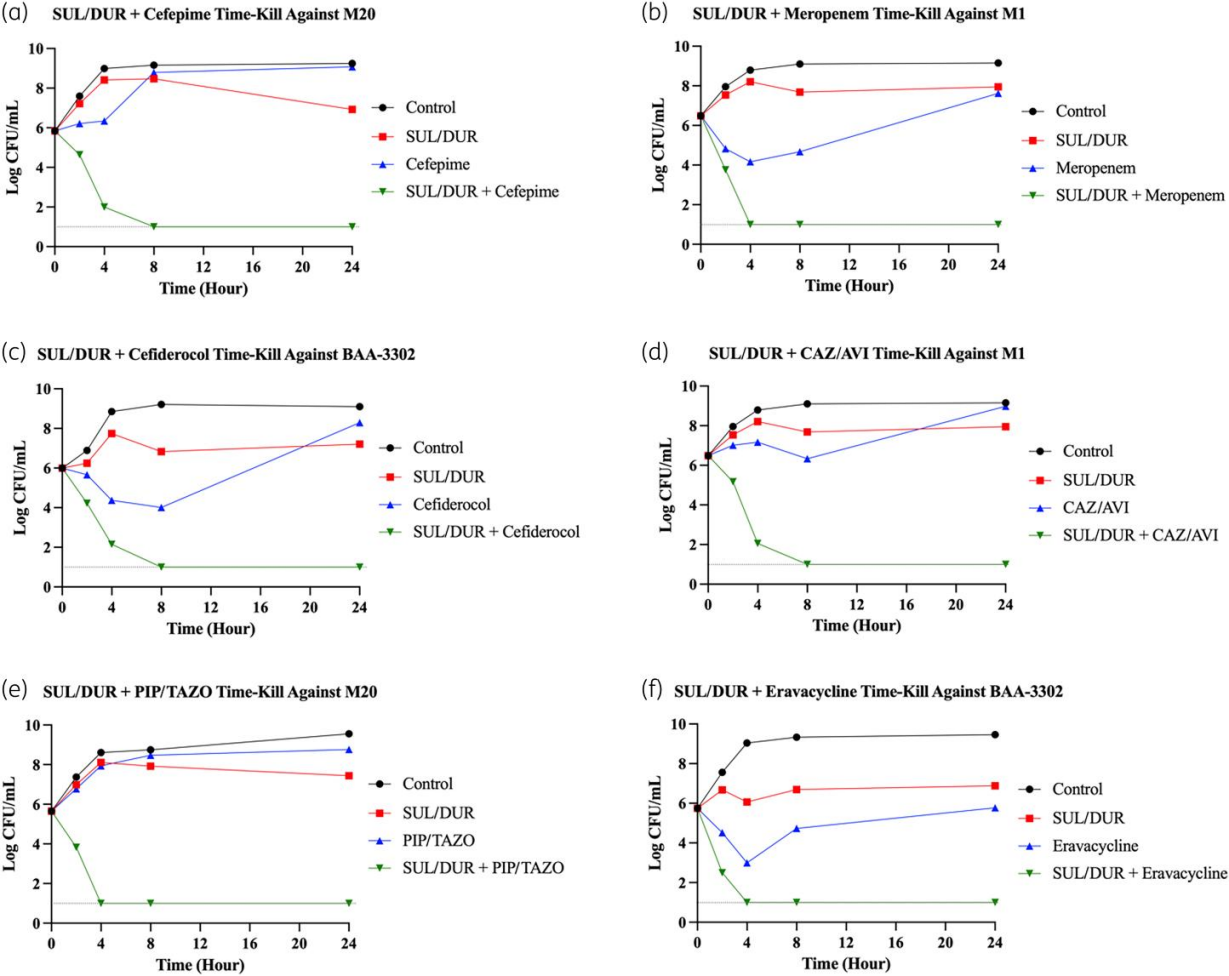
Representative time-kill assay plots showing synergistic bactericidal activity of sulbactam/durlobactam (SUL/DUR) in combination with (a) cefepime, (b) meropenem, (c) cefiderocol, (d) ceftazidime/avibactam (CAZ/AVI), (e) piperacillin/tazobactam (PIP/TAZO), and (f) eravacycline against resistant *A. baumannii* strains, as designated in the figure. Each panel depicts mean log_10_ CFU/mL over 24 h. Each antibiotic was added at 0.5× MIC level for each strain. The grey dashed line represents the lower limit of detection.

Cefepime, meropenem, cefiderocol, ceftazidime/avibactam, and piperacillin/tazobactam each showed potent synergy with sulbactam/durlobactam, achieving rapid and sustained bactericidal synergy (≥2 log reduction compared to the single most active agent) against all three strains, with bacterial counts reaching the lower limit of detection by 4–8 h without regrowth at 24 h (Figures 8a–e). Eravacycline exhibited synergy only against BAA-3302 and additivity against M20, with no interaction observed in M1; a representative plot for BAA-3302 is shown in Figure 8f. Overall, TKA log reductions are summarized in Table 4, and all TKA results are provided in Figures S7–S9 and Table S2.

**Table 4.** Antimicrobial interactions demonstrated by sulbactam/durlobactam in combination with several antibiotics against strains M1, M20, and BAA-3302

Paired antibiotic	Strain	Change in log_10_ CFU/mL at 24 h relative to 0 h	Change from most active single agent after 24 h	Interpretation
Cefepime	M1	**−5**.**49**	**−8**.**16**	**Synergy** ^a^
	M20	**−4**.**84**	**−5**.**92**	**Synergy** ^a^
	BAA-3302	**−5**.**39**	**−3**.**91**	**Synergy** ^a^
Meropenem	M1	**−5**.**49**	**−6**.**63**	**Synergy** ^a^
	M20	**−4**.**84**	**−5**.**93**	**Synergy** ^a^
	BAA-3302	**−5**.**39**	**−6**.**90**	**Synergy** ^a^
Cefiderocol	M1	**−4**.**97**	**−6**.**67**	**Synergy** ^a^
	M20	**−4**.**97**	**−7**.**49**	**Synergy** ^a^
	BAA-3302	**−5**.**00**	**−6**.**21**	**Synergy** ^a^
Ceftazidime/avibactam	M1	**−5**.**49**	**−6**.**95**	**Synergy** ^a^
	M20	**−4**.**84**	**−2**.**00**	**Synergy** ^a^
	BAA-3302	**−4**.**75**	**−5**.**89**	**Synergy** ^a^
Piperacillin/tazobactam	M1	**−4**.**99**	**−5**.**93**	**Synergy** ^a^
	M20	**−4**.**65**	**−6**.**45**	**Synergy** ^a^
	BAA-3302	**−5**.**03**	**−4**.**28**	**Synergy** ^a^
Eravacycline	M1	+1.10	−0.37	Indifferent
	M20	+0.82	−1.57	Additive
	BAA-3302	**−4**.**75**	**−4**.**78**	**Synergy** ^a^

Changes in log_10_ CFU/mL at 24 h relative to baseline (0 h) and the most active single agent are shown, as well as interpretation of the interaction. Synergy was defined as a ≥ 2 log_10_ CFU/mL reduction by the antibiotic combination compared to the most active single agent after 24 h (bold). Additivity was defined as a 1 to <2 log_10_ CFU/mL reduction, and indifference as a < 1 log_10_ CFU/mL reduction under the same conditions.

^a^Synergistic combinations that achieved a reduction in colony counts to the lower limit of detection at 24 h.

## Discussion

As resistance among *A. baumannii* continues to rise, there is an urgent need for new treatment strategies.^2^ Sulbactam/durlobactam, a novel βL/βLI combination recently approved by the FDA for HABP/VABP infections due to susceptible *A. baumannii* isolates, has shown promising efficacy and safety in clinical trials, including reduced nephrotoxicity compared to colistin.^12^ Based on these results, sulbactam/durlobactam is now recommended as first-line therapy for CRAB by the Infectious Diseases Society of America.^13^ In our study, sulbactam/durlobactam was active against all 21 XDR and PDR *A. baumannii* clinical isolates (M1-M22) tested, despite widespread resistance to β-lactam and βL/βLI drugs. Our findings are consistent with recent international surveillance studies reporting potent *in vitro* activity of sulbactam/durlobactam against *A. baumannii*. Karlowsky *et al*. demonstrated that sulbactam/durlobactam inhibited >98% of global *A. baumannii*-*calcoaceticus* complex isolates collected between 2016 and 2021, while Huband *et al*. similarly confirmed high rates of susceptibility in geographically diverse isolates.^23,24^ Together, these data suggest that our results obtained in US clinical isolates are broadly representative of global resistance patterns, although MBL-producing strains remain a critical concern. Although a key limitation of this study is the limited number of strains, these isolates all carried diverse class C and D β-lactamases (e.g. ADCs, OXAs) and in some cases, PBP3 mutations (Table S1). As expected, sulbactam/durlobactam retained activity against all strains, likely due to durlobactam’s broad inhibition of Ambler class A, C, and D enzymes.^14,25^ The only non-susceptible strain tested, BAA-3302 (MIC = 8/4 mg/L), harboured the class B MBLs NDM-1 and L1, which durlobactam does not inhibit.^15^

Synergistic antibiotic combinations may help to prevent or delay the emergence of resistance to sulbactam/durlobactam. A previous study demonstrated *in vitro* synergy between sulbactam/durlobactam and cefepime against *A. baumannii* isolates,^17^ and we previously reported *in vitro* synergy between sulbactam/durlobactam and cefiderocol.^18^ Here, we further evaluated combinations for synergy with a broader panel of antibiotics. Synergy was most consistently observed with β-lactam and βL/βLI agents, including cefepime, meropenem, ceftriaxone, ceftazidime, cefiderocol, ceftazidime/avibactam, and piperacillin/tazobactam, with over 95% of strains exhibiting synergy in chequerboard assays. Except for cefiderocol, these agents are rarely used for the treatment of CRAB infections due to high intrinsic resistance. For example, a large international study of *A. baumannii* isolates found that overall susceptibility rates to cefepime and meropenem were 33.6% and 36.6%, respectively.^26^ Among a large collection of international XDR isolates, the SENTRY Antimicrobial Surveillance Programme reported very low susceptibility rates to cefepime (3.6%), ceftazidime (2.4%), meropenem (8.1%), and piperacillin/tazobactam (0.9%).^26^ Previously, we also showed that our collection of strains was nearly universally resistant to all these individual antibiotics.^19^ We show that the activity of these β-lactam and βL/βLI agents against our collection of *A. baumannii* strains was effectively restored when in combination with sulbactam/durlobactam. These findings were further corroborated in TKAs against select strains, where sulbactam/durlobactam in combination with either cefepime, meropenem, cefiderocol, ceftazidime/avibactam, or piperacillin/tazobactam achieved rapid and sustained bactericidal activity. Notably, synergy was also observed in the MBL-producing strain BAA-3302, although this finding should be considered preliminary given the single isolate evaluated.

The synergistic effect of sulbactam/durlobactam with other β-lactams may be driven by complementary PBP inhibition and restoration of β-lactam activity via durlobactam’s broad β-lactamase inhibition.^17,25^ This synergistic mechanism was sufficient to overcome resistance in BAA-3302, which harbours both MBLs and OXA-type β-lactamases, despite durlobactam's lack of activity against MBLs, suggesting that complementary PBP inhibition and restoration of partner β-lactam activity may be effective even in the presence of MBLs.^15,17^ Even against resistant strains, these combinations achieved strong bactericidal effects *in vitro*, suggesting their clinical potential against highly resistant infections. While we did not assess durlobactam alone, as it is only commercially available with sulbactam, future studies should examine whether it independently enhances the activity of other β-lactam drugs aside from sulbactam.

Among tetracyclines, eravacycline showed the most consistent synergy in chequerboard assays, including against BAA-3302. We previously reported eravacycline, a novel fluorocycline with reported clinical utility against CRAB infections,^27^ to possess relatively potent activity against our collection as monotherapy, as well as synergistic activity in combinations with other β-lactam and βL/βLI agents such as cefepime and ampicillin/sulbactam.^19,28^ However, TKAs demonstrated bactericidal synergy only against BAA-3302, not others, highlighting potential discrepancies between these two synergy detection methods. The enhanced activity of eravacycline, and all other tetracycline drugs tested, in BAA-3302 may be due to sulbactam/durlobactam-mediated altered membrane permeability, allowing increased tetracycline uptake.^29^ BAA-3302 lacks *tet*(*B*), a common tetracycline resistance gene facilitating tetracycline efflux that is widespread in our other strains, which likely contributes to the strain’s high susceptibility to tetracyclines at baseline. However, BAA-3302 does encode multiple RND-type efflux pumps (e.g. *adeABC*, *adeIJK*) that may contribute to differential drug response.^29^ It is uncertain if sulbactam/durlobactam is a substrate of RND-type efflux pumps, but if durlobactam acts as a substrate for RND-type efflux pumps, it may compete with tetracyclines for efflux and thereby enhance intracellular accumulation of the companion agent, which could explain the strong synergy observed.^30^ It is unknown whether such strong synergy between tetracyclines and sulbactam/durlobactam would also be present in MBL-carrying strains with higher baseline tetracycline resistance, and further research is warranted.

A limitation of this work is the lack of pharmacokinetic (PK)/ pharmacodynamic (PD) modelling or *in vivo* validation, which are critical to establishing the clinical relevance of the observed *in vitro* synergy. The antibiotic concentrations used in chequerboard and time-kill assays were selected based on CLSI standards and prior synergy protocols, and time-kill assays were performed at 0.5× MIC to allow for the detection of synergistic interactions. While these conditions are appropriate for mechanistic evaluation, future studies incorporating PK/PD modelling and animal infection models will be important to validate whether the concentrations and synergistic effects observed here can be achieved in clinical settings. Additionally, a key limitation is that only a single MBL-producing *A. baumannii* isolate (BAA-3302) was available for testing. While we observed consistent synergy between sulbactam/durlobactam and several partner antibiotics against this strain, the findings should be considered preliminary. Broader evaluation across diverse MBL-producing isolates is necessary to determine whether these results are generalizable.

Combinations with aminoglycosides, ciprofloxacin, and rifampin showed only modest synergy (20%–40%) in chequerboards. These limited effects may reflect pre-existing resistance or active efflux and warrant further investigation. Strain M11 demonstrated antagonism between sulbactam/durlobactam and rifampin. This antagonism was not evaluated in static time-kill, and chequerboard methods are known to be sensitive to assay conditions. Therefore, we interpret these isolated findings cautiously. Follow-up work using time-kill assays and a larger, diverse strain set will be necessary to determine whether this antagonism is reproducible and clinically meaningful.

### Conclusions

In this study, we evaluated the activity of sulbactam/durlobactam in combination with a broad panel of antibiotics against a collection of highly drug-resistant *A. baumannii* strains, including one carrying MBLs. Our findings demonstrate that sulbactam/durlobactam exhibits potent *in vitro* activity against these isolates, including those harbouring multiple β-lactamases and resistance determinants. When combined with select β-lactam and βL/βLI antibiotics, such as cefepime, meropenem, ceftazidime, cefiderocol, ceftazidime/avibactam, and piperacillin/tazobactam, sulbactam/durlobactam consistently demonstrated strong synergistic effects, restoring activity even in highly resistant backgrounds. These combinations achieved rapid and sustained bactericidal activity in time-kill assays and may represent promising therapeutic strategies to enhance efficacy and suppress resistance development. While our findings are limited to *in vitro* analyses, they strongly support further evaluation of sulbactam/durlobactam-based combinations in PK/PD models, animal infection studies, and ultimately prospective clinical trials to determine their therapeutic utility in patient populations.

## Supplementary Material

dlaf220_Supplementary_Data

## Data Availability

All sequencing data are available on the GeoSeeq platform, a publicly accessible database connecting researchers with tools for public health surveillance (https://portal.geoseeq.com/projects/13774e96-b0c5-41dd-a8f9-43c563cb38d5).

## Supplementary data

Figures S1 to S9 and Tables S1 to S2 are available as Supplementary data at *JAC-AMR* Online.
