# Supplementary material for: Evaluation of sulbactam/durlobactam activity and synergy against highly drug-resistant Acinetobacter baumannii strains
Source: JAC Antimicrob Resist. 2025 Nov 19;7(6):dlaf220. doi: 10.1093/jacamr/dlaf220 (PMC12629082; doi:10.1093/jacamr/dlaf220)
Supplement: dlaf220_Supplementary_Data [file dlaf220_supplementary_data.zip › Halime et al_SUL-DUR_Table S1.pdf]

Strain	MIC	β-Lactam Resistance			Other Resistance					
		OXA	ADC	Other	Aminoglycoside	Tetracycline	Macrolide	Fluoroquinolone	Efflux	Other
M1	2, 4	blaOXA-23, blaOXA-66, blaOXA-94	blaADC-25, blaADC-33, blaADC-94, blaADC-131, blaADC-134, blaADC-194	-	aac(3)-Ia, aadA1, aadA22, ant(3")-IIa, aph(3')-VIa, aph (6)-Id, armA	tet(B), tet(K)	mecA2, mph(E), msr(A)	gyrA_S81L, parC_S84L	-	-
M2	1	blaOXA-1098	blaADC-25	-	ant(3")-IIa	tet(B), tet(K)	-	-	-	abaF, fosLL
M3	1	blaOXA-23, blaOXA-66	blaADC-33	-	ant(3")-IIa, aph(3')-VIa, aph (3")-Ib, armA	tet(B), tet(K)	mecA2, mph(E), msr(E)	-	-	abaF, sul1
M4	1	blaOXA-23, blaOXA-66, blaOXA-423	blaADC-15, blaADC-73, blaADC-145, blaADC-266	fts1_A515V	ant(3")-IIa, aph(3")-Ib, aph (6)-Id, armA	tet(B), tet(K)	mecA2, mph(E), msr(E)	gyrA_S81L, parC_S84L	-	abaF, fosLL, sul1
M5	1	blaOXA-23, blaOXA-66	blaADC-73, blaADC-150, blaADC-256, blaADC-289	fts1_A515V	ant(3")-IIa, aph(3")-Ib, aph (6)-Id, armA	tet(B), tet(K)	mecA2, mph(E), msr(E)	gyrA_S81L, parC_S84L	-	abaF, fosLL
M6	1	blaOXA-23, blaOXA-113	blaADC-335	-	ant(3")-IIa, aph(3')-VIa, aph (3")-Ib, aph(6)-Id, armA	tet(B), tet(K)	mecA2, mph(E), msr(E)	-	-	abaF, fosLL, sul1
M7	0.5	blaOXA-66, blaOXA-1096	blaADC-335, blaADC-337	blaTEM-239, blaR1	aadA1, ant(3")-IIa, aph(3')-Ia, aph(3')-IIIa, aph(3')-Ib, aph(6)-Id, armA	tet(B), tet(K)	mecA2, mph(C), mph(E), msr(E)	-	adeC, amvA	abaF, fosLL, sul1, sul2, catB8, floR
M8	0.5	blaOXA-1098	blaADC-25	-	aadA1, ant(3")-IIa, aph(3')-Ia	tet(B), tet(K)	msr(A)	-	-	fosLL
M9	1	blaOXA-23, blaOXA-51	blaADC-43, blaADC-73, blaADC-110, blaADC-139, blaADC-295, blaADC-326	fts1_A515V	aac(3)-Ia, aadA13, ant(3")-IIa, aph(3')-VIa, aph(3")-Ib, aph(6)-Id, armA	tet(B), tet(K)	mecA2, mph(E), msr(A), msr(E)	gyrA_S81L, parC_S84L	-	abaF, fosLL, sul1
M10	2	blaOXA-66, blaOXA-423,	blaADC-30, blaADC-98, blaADC-133, blaADC-227, blaADC-239, blaADC-423	-	ant(3")-IIa, aph(3")-Ib, aac (3)-Ia, aph(3')-Ia, aph(6)-Id, aadA1, aadA15, aadA22, armA	tet(B)	mph(E)	gyrA_S81L, parC_S84L	adeC, amvA	abaF, sul1, sul2
M11	0.25, 0.5	blaOXA-94, blaOXA-170	blaADC-80, blaADC-148, blaADC-195, blaADC-210, blaADC-256, blaADC-335	-	ant(3")-IIa, aph(3")-Ib, aph (6)-Id, adeC, amvA	tet(B)	msr(E)	gyrA_S81L, parC_E88K	adeC, amvA	dfrB8, sul2, floR
M12	2	blaOXA-23, blaOXA-66	blaADC-337	blaTEM-239	ant(3")-Ia, aph(3")-Ib, aph (3')-Ia, aph(6)-Id, armA	tet(B)	mph(E), msr(E)	-	adeC, amvA	sul2
M13	2	blaOXA-23, blaOXA-202, blaOXA-421, blaOXA-826	blaADC-16, blaADC-150, blaADC-239	-	ant(3")-IIa, aph(3")-Ib, aac (3)-Ia, aph(6)-Id, aadA1, armA	tet(B)	mph(E), msr(E)	parC_S84L	adeC, amvA	abaF, fosLL, dfrB8
M14	1, 2	blaOXA-23, blaOXA-51	blaADC-337	-	ant(3")-IIa, aph(3")-Ib, aph (6)-Id, armA	tet(B)	mph(E), msr(E)	-	adeC, amvA	abaF, sul2
M20	2	blaOXA-23, blaOXA-66, blaOXA-1096	blaADC-15, blaADC-43, blaADC-73, blaADC-131, blaADC-292	fts1_A515V	ant(3")-IIa, aph(3")-Ib, aac (3)-Ia, aph(6)-Id	tet(B)	mph(E), msr(E)	gyrA_S81L, parC_S84L	adeC, amvA	abaF, sul1, sul2
BAA-3302	8	blaOXA-94	-	blaNDM-1, blaL1	ant2-Ia, ant(3')-II, aph3-VI	-	mph(D), msr(E)	-	adeABC, adeFGH, adeIJK, adeL, adeN, adeRS, baeSR, emrB, msbA, tolC, soxR	catB, rlmN, sul2
