# Supplementary material for: Evaluation of sulbactam/durlobactam activity and synergy against highly drug-resistant Acinetobacter baumannii strains
Source: JAC Antimicrob Resist. 2025 Nov 19;7(6):dlaf220. doi: 10.1093/jacamr/dlaf220 (PMC12629082; doi:10.1093/jacamr/dlaf220)
Supplement: dlaf220_Supplementary_Data [file dlaf220_supplementary_data.zip › Halim_SUL-DUR_SupplementaryFigures_Update.docx]

**Evaluation of Sulbactam/Durlobactam Activity and Synergy Against Highly Drug-Resistant *Acinetobacter baumannii* Strains**

Justin Halim, Jeannete Bouzo, and Valerie J. Carabetta

**Table of Contents**

[**Figure S1.** Representative checkerboard assay with sulbactam/durlobactam and ceftazidime against strain M20. 2](#_Toc208987416)

[**Figure S2.** Representative checkerboard assay with sulbactam/durlobactam and piperacillin/tazobactam against strain M20. 3](#_Toc208987417)

[**Figure S3.** Representative checkerboard assay with sulbactam/durlobactam and ceftriaxone against strain M20. 4](#_Toc208987418)

[**Figure S4.** Representative checkerboard assay with sulbactam/durlobactam and cefiderocol against strain M1. 5](#_Toc208987419)

[**Figure S5.** Representative checkerboard assay with sulbactam/durlobactam and omadacycline against strain M22. 6](#_Toc208987420)

[**Figure S6.** Representative checkerboard assay with sulbactam/durlobactam and rifampin against strain M11. 7](#_Toc208987421)

[**Figure S7.** Time-kill assay plots of log_10_ CFU/mL vs. time for antibiotic combinations against strain M1. 8](#_Toc208987422)

[**Figure S8.** Time-kill assay plots of log_10_ CFU/mL vs. time for antibiotic combinations against strain M20. 9](#_Toc208987423)

[**Figure S9.** Time-kill assay plots of log_10_ CFU/mL vs. time for antibiotic combinations against strain BAA-3302. 10](#_Toc208987424)

# **Figure S1.** Representative checkerboard assay with sulbactam/durlobactam and ceftazidime against strain M20.


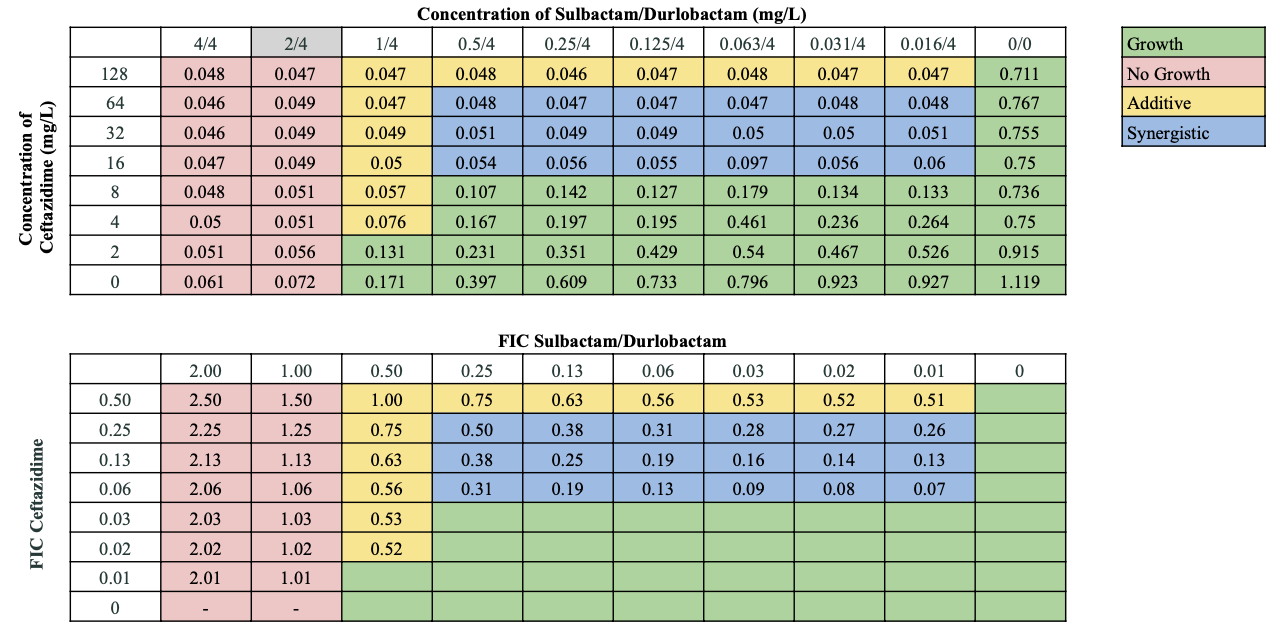


Top: OD_600_ measurements following 16 hours of static growth at 37°C. The MIC values for each drug alone are highlighted. If the MIC value exceeded the initial concentration, then no value is highlighted. No bacterial growth occurred in wells where OD_600_ <0.1, and above this cutoff, bacterial growth did occur. Bottom: Fractional inhibitory concentration (FIC) values were calculated for each drug (concentration/MIC) and added together for all wells where no growth was observed. Additive interactions (FICI between 0.5-1.0), and synergistic interactions (FICI ≤0.5) are indicated.

# **Figure S2.** Representative checkerboard assay with sulbactam/durlobactam and piperacillin/tazobactam against strain M20.

**
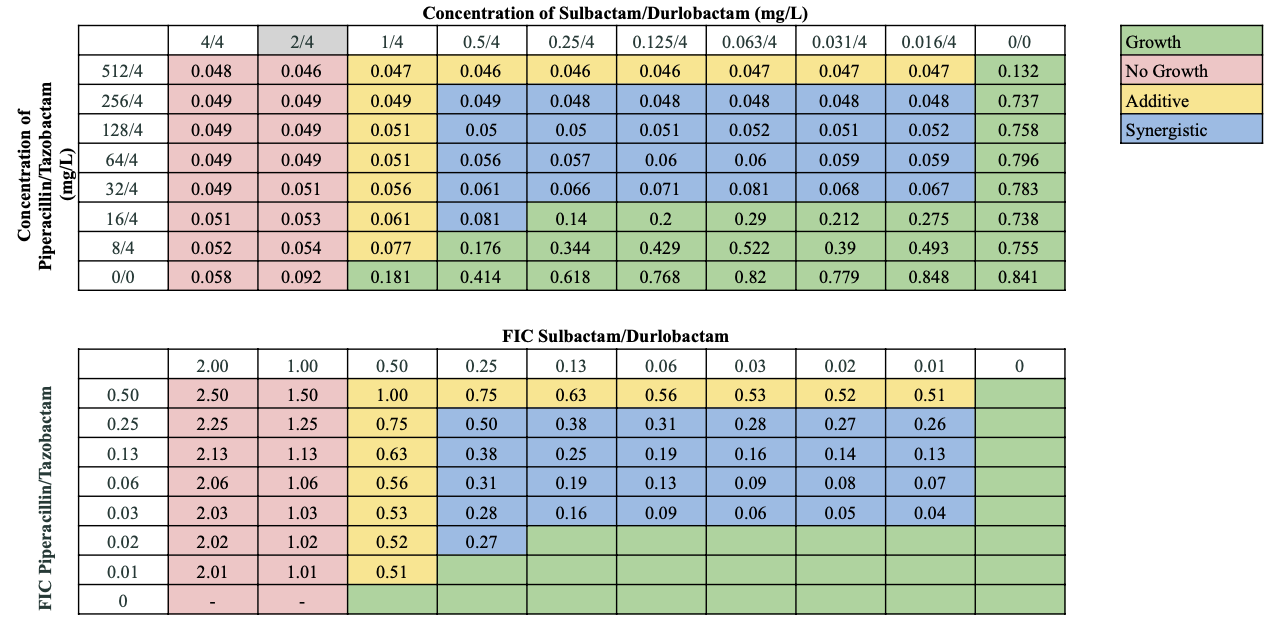
**

Top: OD_600_ measurements following 16 hours of static growth at 37°C. The MIC values for each drug alone are highlighted in gray. If the MIC value exceeded the initial concentration, then no value is highlighted. No bacterial growth occurred in wells where OD_600_ <0.1, and above this cutoff, bacterial growth did occur. Bottom: Fractional inhibitory concentration (FIC) values were calculated for each drug (concentration/MIC) and added together for all wells where no growth was observed. Additive interactions (FICI between 0.5-1.0), and synergistic interactions (FICI ≤0.5) are indicated.

# **Figure S3.** Representative checkerboard assay with sulbactam/durlobactam and ceftriaxone against strain M20.

**
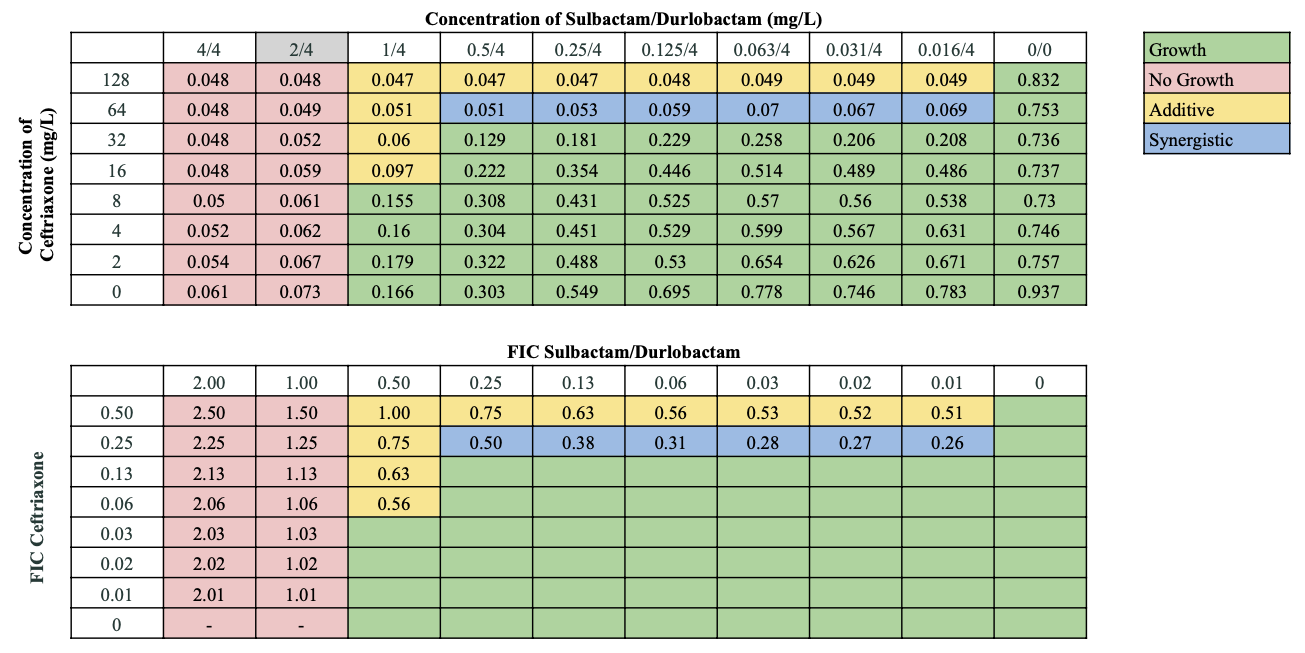
**

Top: OD_600_ measurements following 16 hours of static growth at 37°C. The MIC values for each drug alone are highlighted in gray. If the MIC value exceeded the initial concentration, then no value is highlighted. No bacterial growth occurred in wells where OD_600_ <0.1, and above this cutoff, bacterial growth did occur. Bottom: Fractional inhibitory concentration (FIC) values were calculated for each drug (concentration/MIC) and added together for all wells where no growth was observed. Additive interactions (FICI between 0.5-1.0), and synergistic interactions (FICI ≤0.5) are indicated.

# **Figure S4.** Representative checkerboard assay with sulbactam/durlobactam and cefiderocol against strain M1.


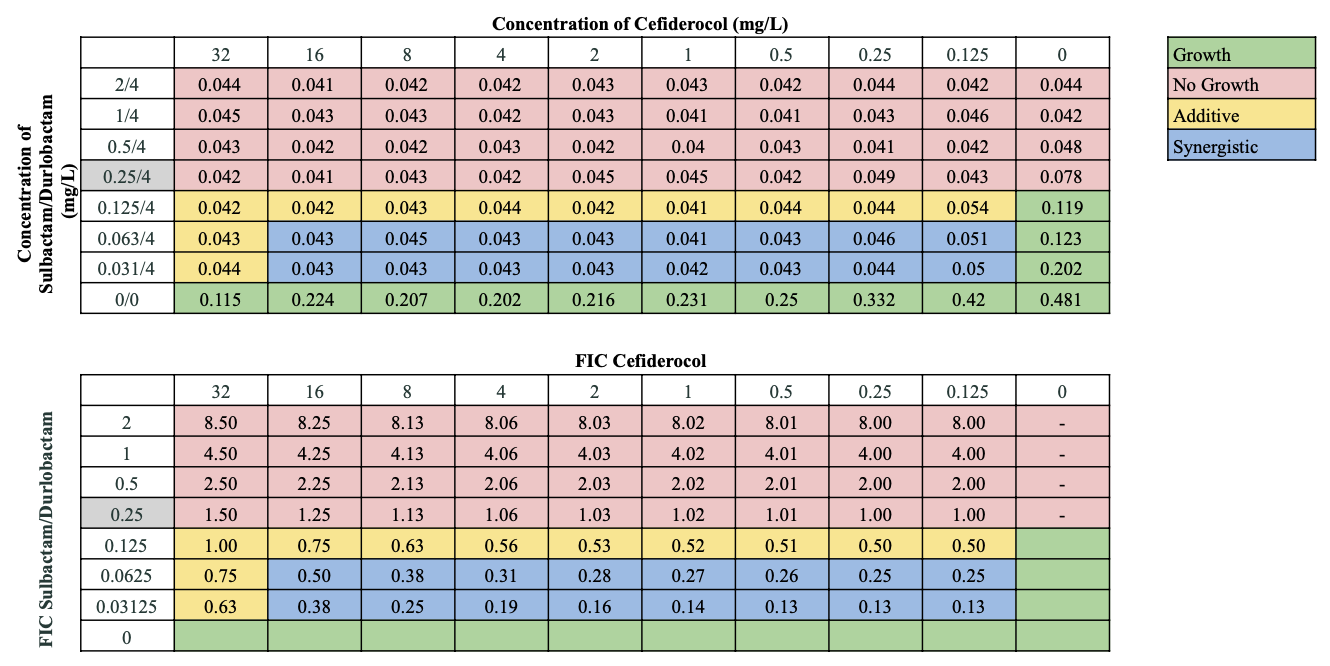


Top: OD_600_ measurements following 16 hours of static growth at 37°C. The MIC values for each drug alone are highlighted in gray. If the MIC value exceeded the initial concentration, then no value is highlighted. No bacterial growth occurred in wells where OD_600_ <0.1, and above this cutoff, bacterial growth did occur. Bottom: Fractional inhibitory concentration (FIC) values were calculated for each drug (concentration/MIC) and added together for all wells where no growth was observed. Additive interactions (FICI between 0.5-1.0), and synergistic interactions (FICI ≤0.5) are indicated. Note sulbactam/durlobactam is diluted vertically and cefiderocol horizontally.

# **Figure S5.** Representative checkerboard assay with sulbactam/durlobactam and omadacycline against strain M22.


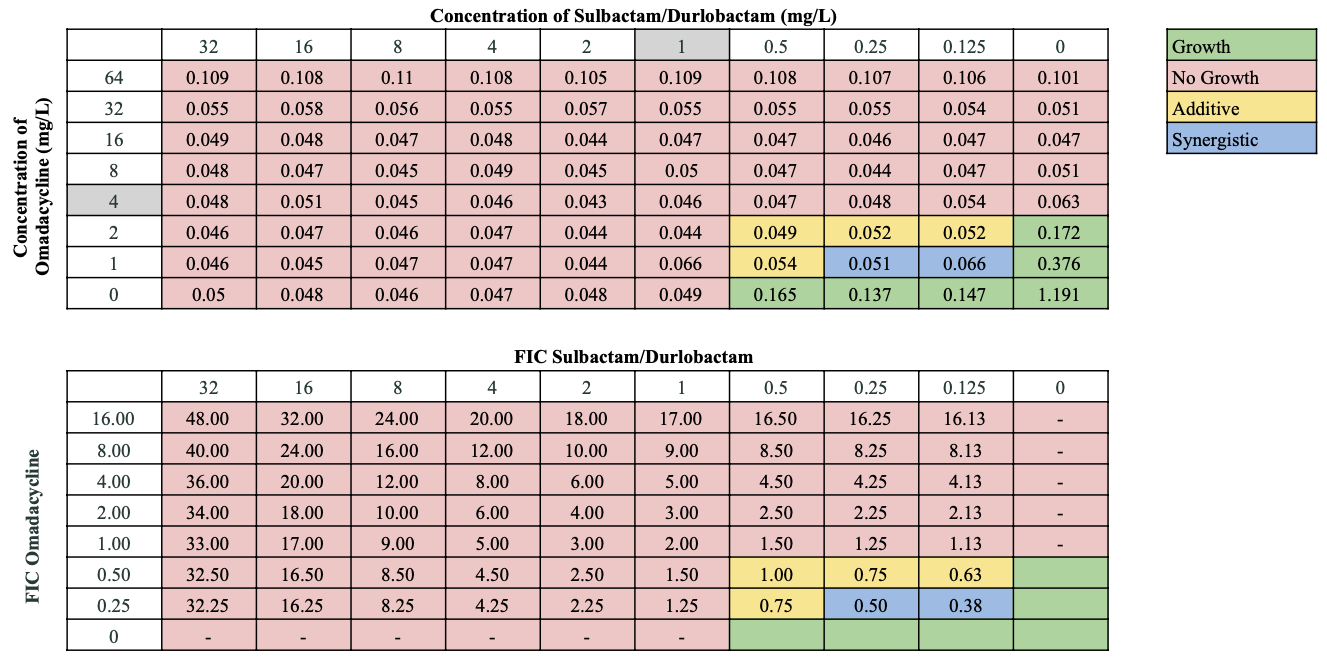


Top: OD_600_ measurements following 16 hours of static growth at 37°C. The MIC values for each drug alone are highlighted in gray. No bacterial growth occurred in wells where OD_600_ <0.1, and above this cutoff, bacterial growth did occur. Bottom: Fractional inhibitory concentration (FIC) values were calculated for each drug (concentration/MIC) and added together for all wells where no growth was observed. Additive interactions (FICI between 0.5-1.0), and synergistic interactions (FICI ≤0.5) are indicated. Note sulbactam/durlobactam was added at an initial concentration of 32/4 mg/L rather than 4/4 mg/L.

# **Figure S6.** Representative checkerboard assay with sulbactam/durlobactam and rifampin against strain M11.


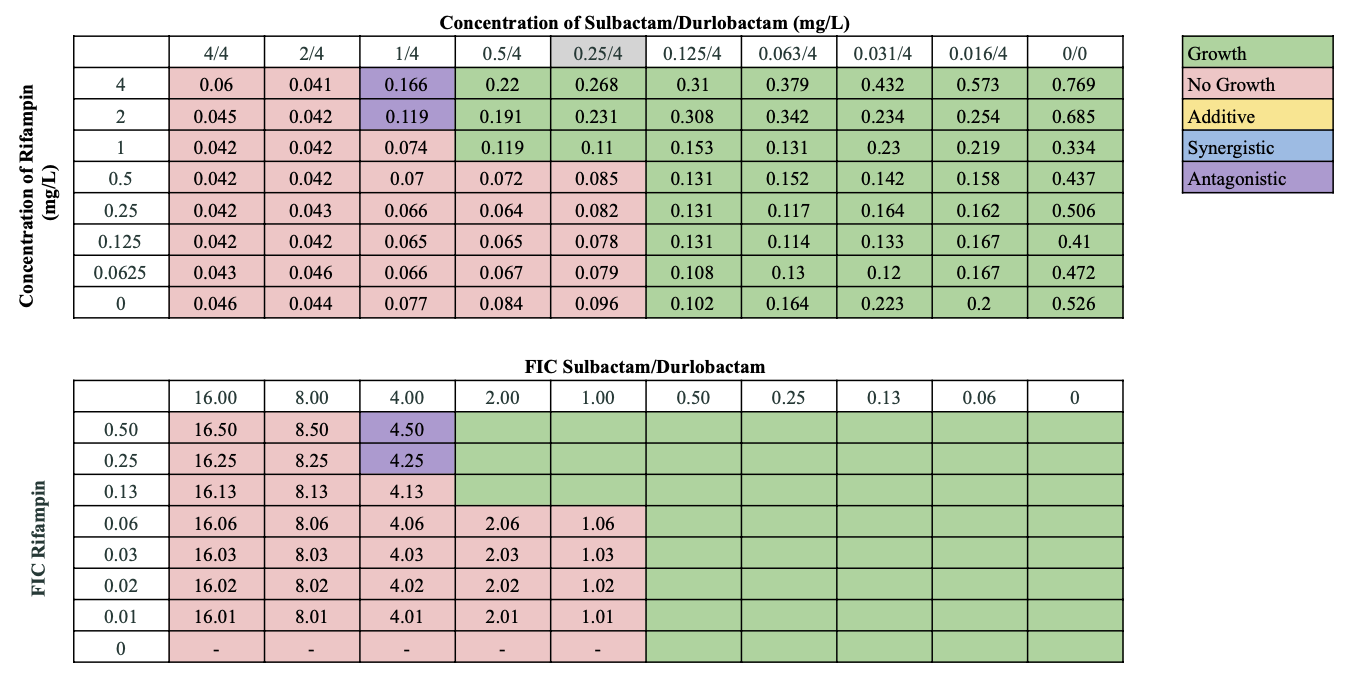


Top: OD_600_ measurements following 16 hours of static growth at 37°C. The MIC values for each drug alone are highlighted in gray. If the MIC value exceeded the initial concentration, then no value is highlighted. No bacterial growth occurred in wells where OD_600_ <0.1, and above this cutoff, bacterial growth did occur. Bottom: Fractional inhibitory concentration (FIC) values were calculated for each drug (concentration/MIC) and added together for all wells where no growth was observed. Additive interactions (FICI between 0.5-1.0), and synergistic interactions (FICI ≤0.5) are indicated. Potential antagonistic interactions (FICI >4.0) are indicated. Note that at lower concentrations of rifampin at 1/4 sulbactam/durlobactam, there is no growth, suggesting antagonism at these concentrations.

# **Figure S7.** Time-kill assay plots of log_10_ CFU/mL vs. time for antibiotic combinations against strain M1.

**
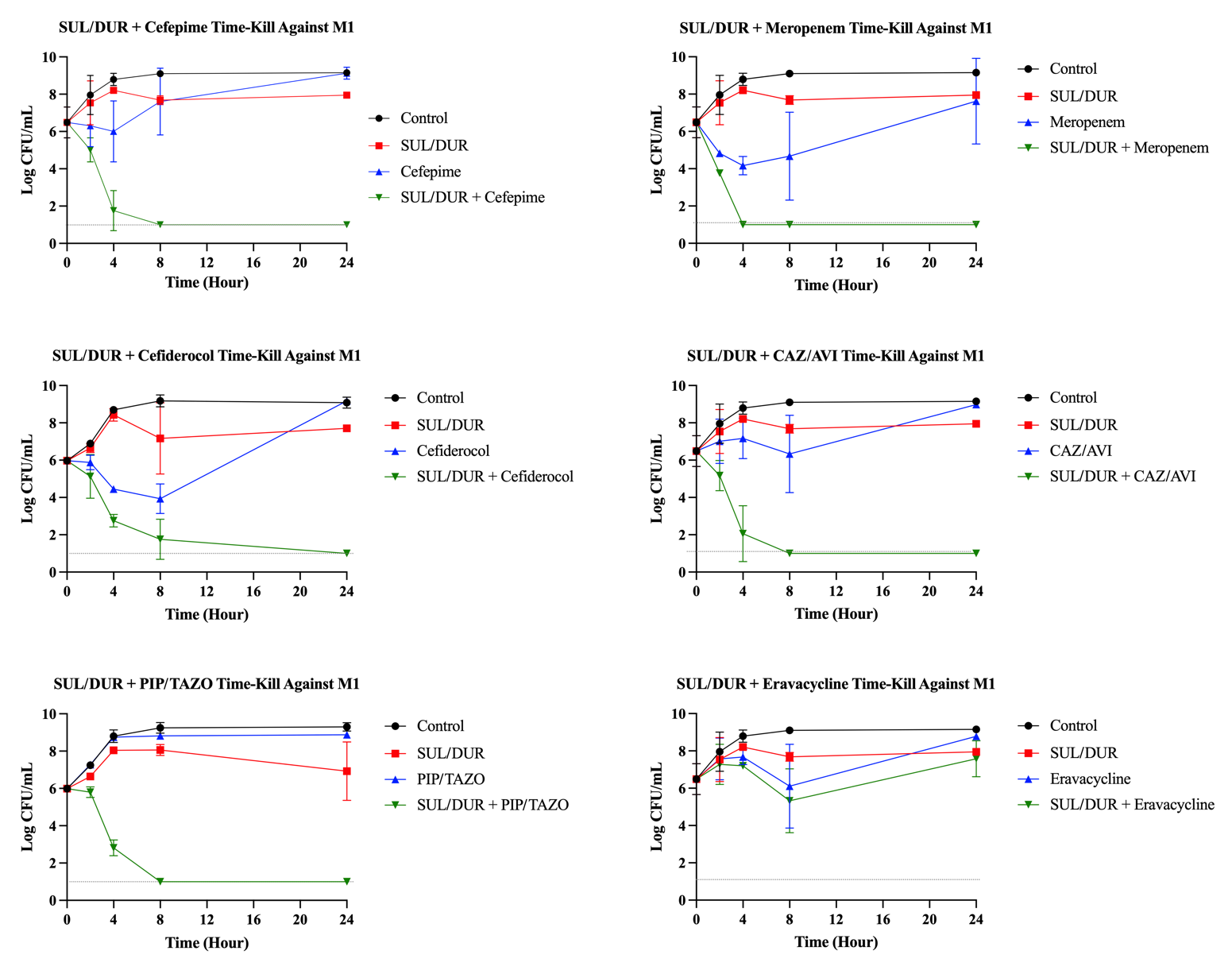
**

The left panel in descending order shows sulbactam/durlobactam (SUL/DUR) in combination with cefepime, cefiderocol, and piperacillin/tazobactam (PIP/TAZO), while the right panel in descending order shows sulbactam/durlobactam in combination with meropenem, ceftazidime/avibactam (CAZ/AVI), and eravacycline. All antibiotics were added at 0.5x MIC level. The dashed grey line represents the lower limit of detection. Error bars are shown and represent standard deviation of the mean. All determinations are averages of technical triplicates from two independent determinations.

# **Figure S8.** Time-kill assay plots of log_10_ CFU/mL vs. time for antibiotic combinations against strain M20.

**
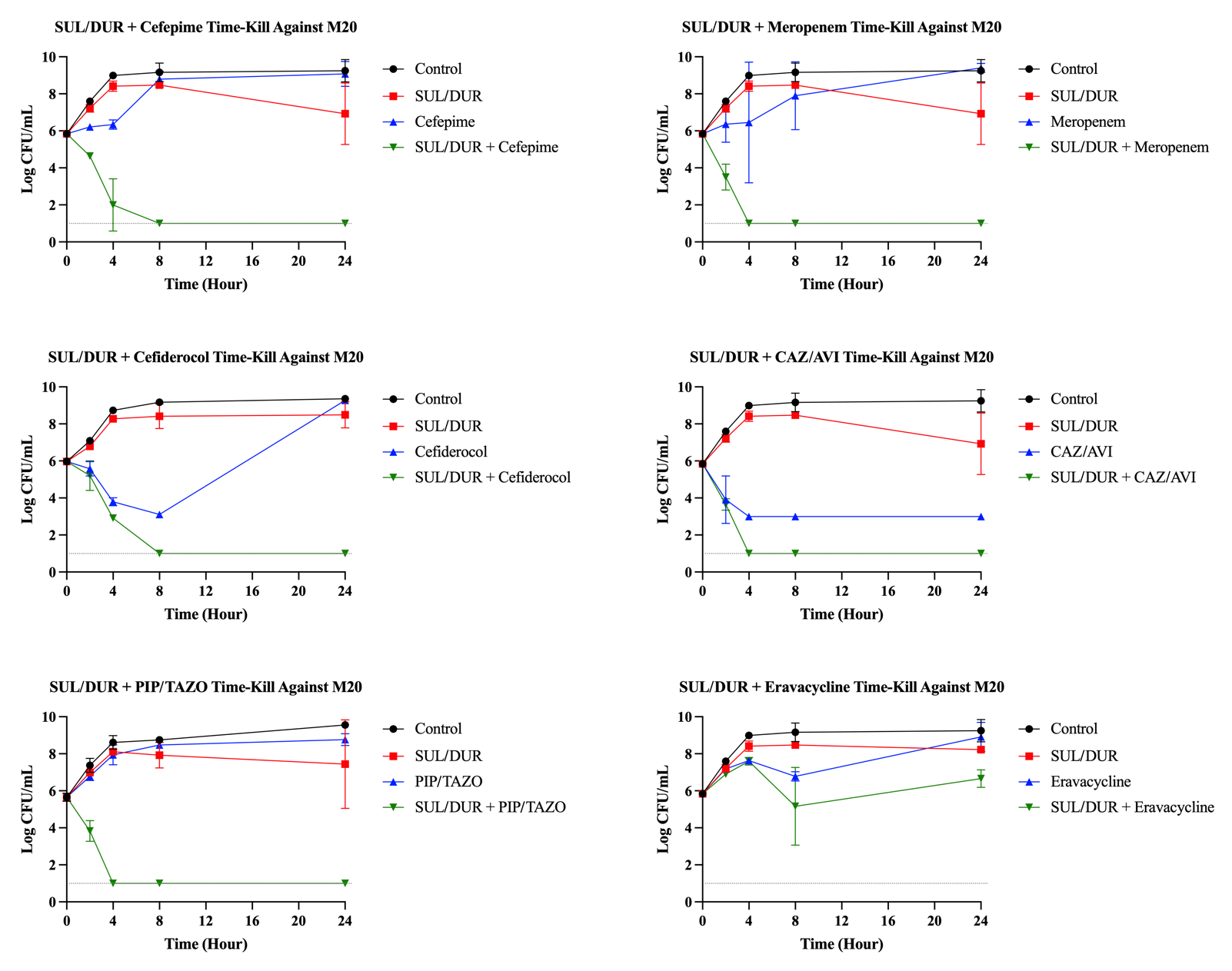
**

The left panel in descending order shows sulbactam/durlobactam (SUL/DUR) in combination with cefepime, cefiderocol, and piperacillin/tazobactam (PIP/TAZO), while the right panel in descending order shows sulbactam/durlobactam in combination with meropenem, ceftazidime/avibactam (CAZ/AVI), and eravacycline. All antibiotics were added at 0.5x MIC level. The dashed grey line represents the lower limit of detection. Error bars are shown and represent standard deviation of the mean. All determinations are averages of technical triplicates from two independent determinations.

# **Figure S9.** Time-kill assay plots of log_10_ CFU/mL vs. time for antibiotic combinations against strain BAA-3302.

**
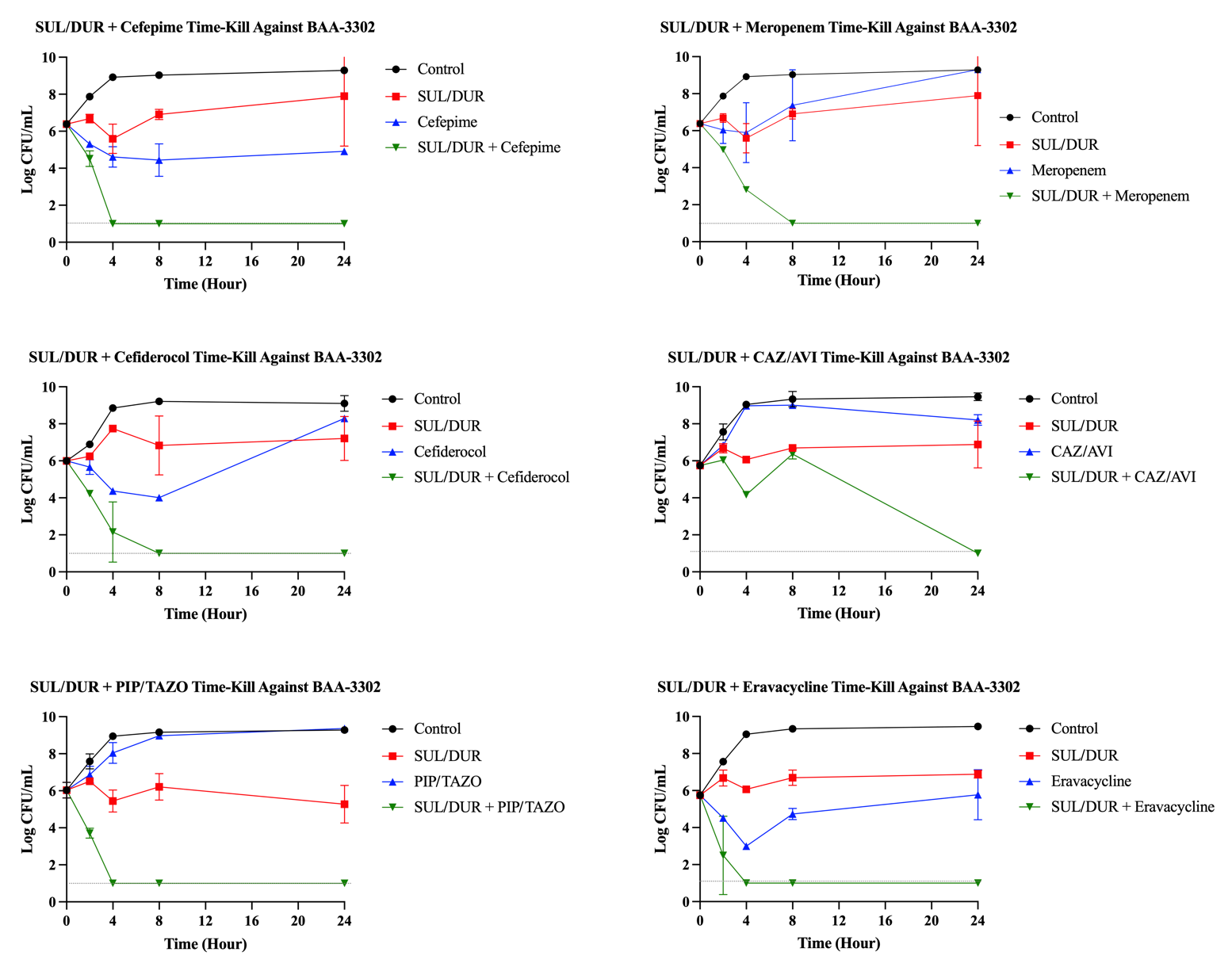
**

The left panel in descending order shows sulbactam/durlobactam (SUL/DUR) in combination with cefepime, cefiderocol, and piperacillin/tazobactam (PIP/TAZO), while the right panel in descending order shows sulbactam/durlobactam in combination with meropenem, ceftazidime/avibactam (CAZ/AVI), and eravacycline. All antibiotics were added at 0.5x MIC level. The dashed grey line represents the lower limit of detection. Error bars are shown and represent standard deviation of the mean. All determinations are averages of technical triplicates from two independent determinations.
